# Quantitative Characteristics of Toxic Compounds According to the Solvent Type

**DOI:** 10.1155/2019/3201370

**Published:** 2019-04-30

**Authors:** Young-Ji An, Seong-Jin Choi, Yong-Hyun Kim, Kyuhong Lee

**Affiliations:** ^1^Department of Toxicology Evaluation, Konyang University, Daejeon, Republic of Korea; ^2^Jeonbuk Department of Inhalation Research, Korea Institute of Toxicology, Jeongeup 56212, Republic of Korea; ^3^Human and Environmental Toxicology, University of Science and Technology, Daejeon 34113, Republic of Korea; ^4^National Center for Efficacy Evaluation of Respiratory Disease Product, Korea Institute of Toxicology, Jeongeup 56212, Republic of Korea

## Abstract

The quantitative analysis of target substances is an important part of assessing the toxicity of diverse materials. Usually, the quantitation of target compounds is conducted by instrumental analysis such as chromatography and capillary electrophoresis. If solvents are used in the pretreatment step of the target analyte quantification, it would be crucial to examine the solvent effect on the quantitative analysis. Therefore, in this study, we assessed the solvent effects using four different solvents (methanol, hexane, phosphate buffered saline (PBS), and dimethyl sulfoxide (DMSO)) and three toxic compounds (benzene, toluene, and methylisothiazolinone (MIT)). Liquid working standards containing the toxic compounds were prepared by dilution with each solvent and analyzed by gas chromatography-mass spectrometry (GC-MS). As a result, we found that the response factor (RF) values of the target analytes were different, depending on the solvent types. In particular, benzene and toluene exhibited their highest RF values (33,674 ng^−1^ and 78,604 ng^−1^, respectively) in hexane, while the RF value of MIT was the highest (9,067 ng^−1^) in PBS. Considering the correlation (*R*^2^) and relative standard deviation (RSD) values, all target analytes showed fairly good values (*R*^2^ > 0.99 and RSD < 10%) in methanol and DMSO. In contrast, low *R*^2^ (0.0562) and high RSD (10.6%) values of MIT were detected in hexane, while benzene and toluene exhibited relatively low *R*^2^ and high RSD values in PBS (mean *R*^2^ = 0.9892 ± 0.0146 and mean RSD = 13.3 ± 4.1%). Based on these findings, we concluded that the results and reliability of the quantitative analysis change depending on the analyte and solvent types. Therefore, in order to accurately assess the toxicity of target compounds, reliable analytical data should be obtained, preferentially by considering the solvent types.

## 1. Introduction

Chemical products that are generally used to clean, sanitize, and disinfect are widely employed in our living environments. However, several of these are known to contain toxic compounds, which can damage the human health and natural environment [1, 2]. Due to their hazardous properties, these chemicals are usually regulated through a toxicology analysis such as safety assessment and toxicity testing [3]. In the toxicity testing, the accurate exposure of target chemicals to experimental animals or cells is important. As such, the stability of target chemicals should first be evaluated and ensured, the latter of which is commonly performed with the use of solvents [4, 5]. In case of the U.S. Environmental Protection Agency, the use of acetone, dimethylformamide (DMF), dimethyl sulfoxide (DMSO), and methanol as solvents is recommended for toxicity tests on aquatic invertebrates [6].

In general, a variety of solvents have been used to performing toxicity test [7, 8]. For example, in the case of an inhalation toxicity testing, solvents are mainly used for the sample extraction, absorption, and dilution steps. More specifically, if the filter sampling is conducted, the inhalable samples are first collected by filters (i.e., glass, quartz, or Teflon filters), after which the samples loaded on the filters are extracted and diluted by solvents [9–12]. Solvents are also used as the absorption solution to collect directly the inhalable samples [13]. In contrast, in the case of a cytotoxicity test, solvents are used for the storage and extraction of target analytes from cells [14, 15]. For this purpose, phosphate buffered saline (PBS) has been recommended as a suitable buffer solution to maintain the appropriate pH for cell storage [16, 17]. In addition, DMSO can be used as a sample extractant and cryoprotectant of cultured cells in biochemistry and cell biology [18].

Reportedly, the calibration results of target compounds can differ depending on the solvent effect due to the use of liquid samples [19, 20]. For example, Campos et al. [21] examined the reaction of an imidazole derivative with organic solvents by analyzing the imidazole samples derivatized from diethyl 2,4-dinitrophenyl phosphate and DMSO or distilled water as a solvent. As a result, they found that the imidazole derivative reaction increases in DMSO and decreases in distilled water, which indicates that the reactivity of derivation is different depending on the solvent type and that the reliability results can affect the quantitative analysis.

In this study, we investigated the solvent effect of toxic compounds in relation to their calibration characteristics. Methanol (MeOH), hexane, PBS, and DMSO, which are commonly used in the chemical and biological analysis, were selected as target solvents, while benzene, toluene, and methylisothiazolinone (MIT) were selected as target analytes (Table 1). From these, benzene and toluene are generally identified as carcinogenic, as they have the potential to damage the generative functions in humans upon transmission [22], whereas MIT is commonly known as the main component in humidifier disinfectants. The standard solutions containing the target analytes and solvents were analyzed using a gas chromatography (GC) equipped with a mass spectrometry (MS), which provided the calibration data to assess the solvent effects.

## 2. Materials and Methods

### 2.1. Preparation of the Working Standards (WSs)

**Figure 1:**
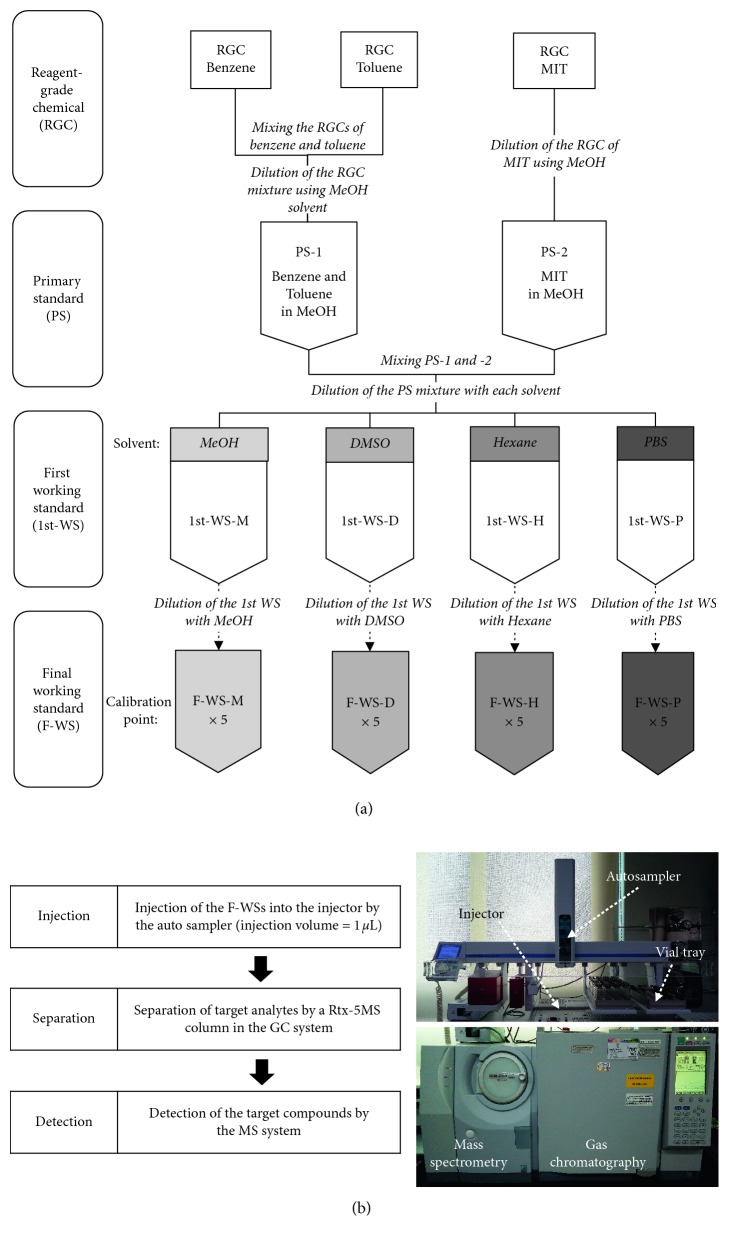
A plot of the experimental sequence for the preparation and analysis of the working standards (WSs). (a) Preparation of working standards. (b) Analysis of the working standards.

A total of three target compounds (benzene, toluene, and MIT) and four solvents (MeOH, hexane, PBS, and DMSO) were selected to investigate the solvent effect. WSs of these three target compounds were prepared in the same way using each solvent. Reagent grade chemicals (RGCs) were purchased at ≥95% purity: (1) 99.5% (benzene and toluene), (2) 95% (MIT), and (3) 99.9% (MeOH, hexane, and DMSO) (Sigma-Aldrich, USA). PBS (1x, pH 7.4, Gibco BRL) was purchased from Life Technologies (Frederick, MD, USA). Primary standards (PSs) were prepared (PS-1 and PS-2) by mixing the RGCs with benzene and toluene or with MIT, respectively, with concentrations of 8,736 ng·*μ*L^−1^ (benzene), 8,627 ng·*μ*L^−1^ (toluene), and 90,000 ng·*μ*L^−1^ (MIT). The first working standards (1st-WSs) were prepared by mixing 100 *μ*L of PS-1 and PS-2 each and 1800 *μ*L of the respective solvent in a 2 mL vial, resulting in final concentrations (ng·*μ*L^−1^) of 416 (benzene), 411 (toluene), and 4,286 (MIT). Four different solvents were used to form the 1st-WSs: MeOH (1st-WS-M), hexane (1st-WS-H), PBS (1st-WS-P), and DMSO (1st-WS-D). The final working standards (F-WS-M, F-WS-H, F-WS-P, and F-WS-D) for the five-point calibrations were prepared by diluting each 1st-WS with the respective solvent to prepare five different concentrations: (1) benzene: 8.32, 20.8, 41.6, 83.2, and 208 ng·*μ*L^−1^, (2) toluene: 8.22, 20.5, 41.1, 82.2, and 205 ng·*μ*L^−1^, and (3) MIT: 85.7, 214, 429, 857, and 2,143 ng·*μ*L^−1^ (Figure 1(a)). Detailed information on the preparation of the WSs is shown in Table 2.

### 2.2. Instrumental System

A GC (GC-2010, Shimadzu, Japan) equipped with an MS (GCMS-QP2010 ultra, Shimadzu, Japan) was employed to quantify the toxic compounds in the solvents (Figure 1(b)). Since the F-WSs contained different solvents, the calibration characteristics of the toxic compounds according to the solvent effects were assessed.

In the analysis, 1 *µ*L samples from the F-WSs were injected into the GC injector (at 250°C) using the autosampler (AOC-5000, Shimadzu, Japan). The target analytes were then transferred to the Rtx-5MS column (diameter: 0.25 mm, length: 60 m, and thickness: 0.25 *µ*m, Restek Corporation, USA) for separation using a carrier gas (He > 99.999%, flow rate of 2.41 mL·min^−1^ (constant flow)). The oven temperature of the GC was initially set to 40°C for 4 min, after which it was ramped at 15°C·min^−1^ to 145°C, and finally ramped at 70°C·min^−1^ to 285°C, thereby giving a total run time of 13 min.

The target analytes separated by the GC system were then detected by the MS system. Both the interface and ion source temperatures were set to 250°C. The target analytes were quantified in a total ion chromatogram (TIC) mode in a mass range of 30–500 m/z. Extracted ion chromatographic (EIC) mode was subsequently applied to the minimized interfaces using significant ions identified from the spectrum of each target analyte (Table 1). Detailed setting information of the analysis instrument is presented in Table 3.

## 3. Result and Discussion

### 3.1. Calibration Characteristics of the Toxic Compounds According to the Solvent Type

The calibration results of the target analytes obtained by GC-MS analysis were provided in terms of the response factor (RF, ng^−1^), coefficient of determination (*R*^2^), relative standard error (RSD, %), and limit of detection (LOD, ng) (Table 4).

Notably, the RF value of each target analyte was different, depending on the solvent type. The obtained RF values were normalized against the highest RF value in the following way: normalized-RF (N-RF) = RF/RF(max) (Table 4). Benzene and toluene had relatively high N-RF values (above 0.77) in hexane and DMSO, while in PBS (highly polar solvent), their N-RF values were significantly lower (0.34 and 0.27, respectively). In contrast, MIT exhibited the highest N-RF value in PBS (N-RF = 1), whereas in hexane, the N-RF value of MIT was low (N-RF = 0.12).

**Figure 2:**
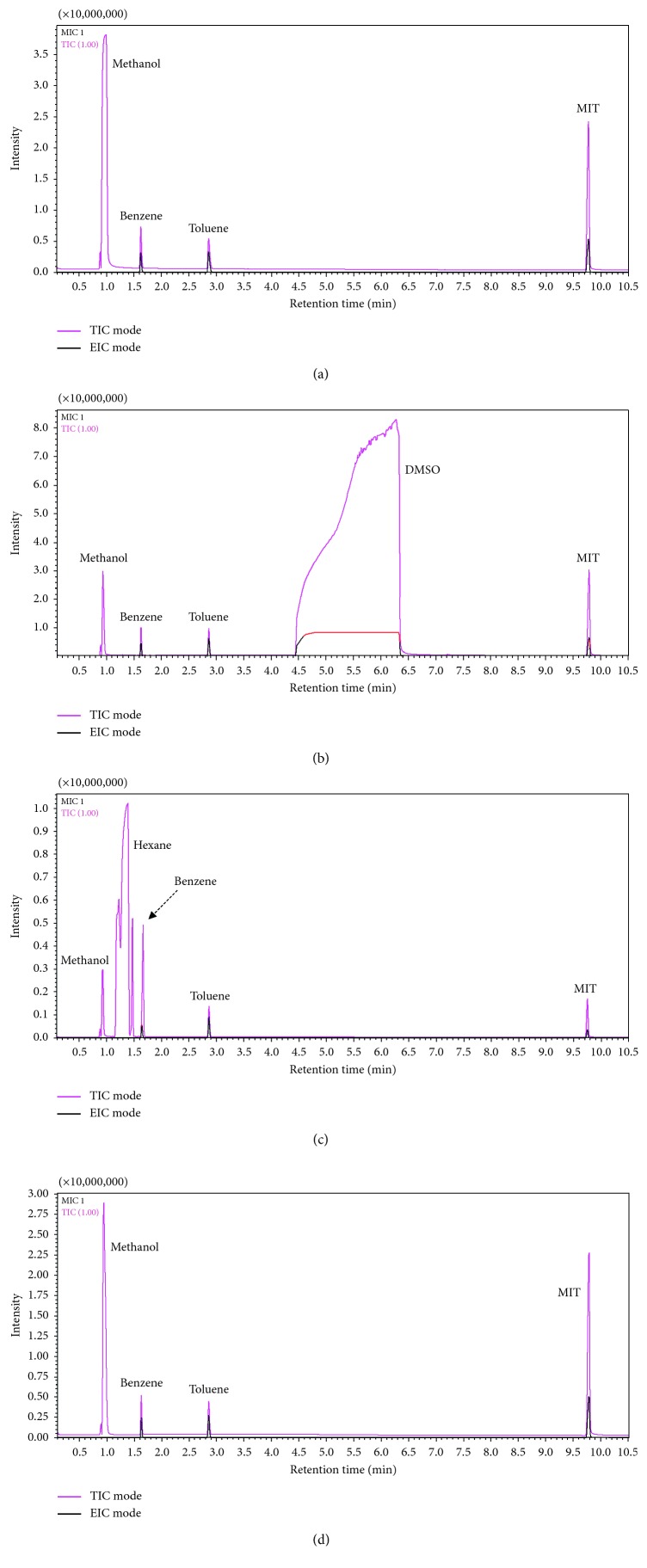
Chromatograms of the three target compounds based on four different solvents. The concentrations varied 208 ng·*μ*L^−1^ (benzene), 205 ng·*μ*L^−1^ (toluene), and 2,143 ng·*μ*L^−1^ (MIT). (a) MeOH solvent. (b) DMSO solvent. (c) Hexane solvent. (d) PBS solvent.

The calibration results derived in terms of *R*^2^ and RSD (%) were similar to the patterns observed in the RF values (Figure 2). In particular, the *R*^2^ values of benzene, toluene, and MIT in MeOH and DMSO were fairly high (>0.99). In MeOH, the RSD values of all target compounds exhibited the best reproducibility (below 6% for all target analytes), while in DMSO, the RSD values were slightly higher (mean RSD (*n*=3) = 8.09 ± 2.13%). When PBS and hexane were used as solvents, the *R*^2^ and RSD values of the target compounds differed upon changing the solvent types. In hexane, the *R*^2^ values of benzene and toluene exhibited a strong linearity (>0.96), while that of MIT was low (0.0562). Also, benzene and toluene showed good RSD values in hexane (<5%), whereas MIT had a high RSD (10.6%). In contrast, the RSD and *R*^2^ values when PBS was used as a solvent were contrary to those obtained in the case of hexane. In particular, the *R*^2^ and RSD values of MIT in PBS were 0.9997 and 2.41%, respectively, while the RSD values of benzene and toluene showed low reproducibility with above 14%. The LOD values of all target analytes were below 0.18 ng, which is sufficient to detect the lowest calibration points of all the types of final working standards (F-WSs) (Table 4).

Based on these results, we concluded that the instrument responsivity and reproducibility of the target compounds differed, depending on their physicochemical properties. Moreover, the responsivity and analytical reliability was found to be especially affected by the solvent type. Therefore, in order to achieve an accurate quantitation, it is important to select the solvent by considering the physicochemical properties (i.e., polarity) of the target analytes.

### 3.2. Comparison of Previous Research Data

Diverse solvents have been previously used in chemical and biological analyses for the pretreatment of target samples and the preparation of standard solutions. In this study, we confirmed that the calibration results were different depending on the solvent type, although the same target compounds were analyzed by the same analytical methods. However, many researchers do not fully consider the solvent effect in their quantitative analyses (Table 5).

For example, Rezende et al. [24] analyzed formaldehyde in bovine milk using high-performance liquid chromatography-ultraviolet/visible (HPLC-UV) by employing ultrapure water as a standard solvent and acetonitrile as a sample solvent. Moreover, Baümler et al. [28] analyzed sugars in sunflower oil samples using HPLC by extracting them with ethanol and subsequently diluting the samples with distilled water. Additionally, a 0.005 N H_2_SO_4_ solution was used as a standard solvent. In both of these cases, there could be a quantitative error due to the solvent difference between the standards and samples.

Furthermore, there are also studies that use the same solvent for both standard and sample preparation. For instance, Lim et al. [25] analyzed ferrocyanide ions using HPLC-UV by employing the same solvent (0.02 M NaOH solution) for the preparation of the standard solution and pretreatment of the sample. In addition, Klimczak and Gliszczyńska-Świgło [27] quantified vitamin C (ascorbic acid and dehydroascorbic acid) using HPLC and ultraperformance liquid chromatography (UPLC) systems by using 10% meta-phosphoric acid solvent to both extract the samples and dilute the standard solutions. In both of these cases, the solvent effects could be minimized by using the same solvent for the quantitative analysis.

## 4. Conclusion

**Figure 3:**
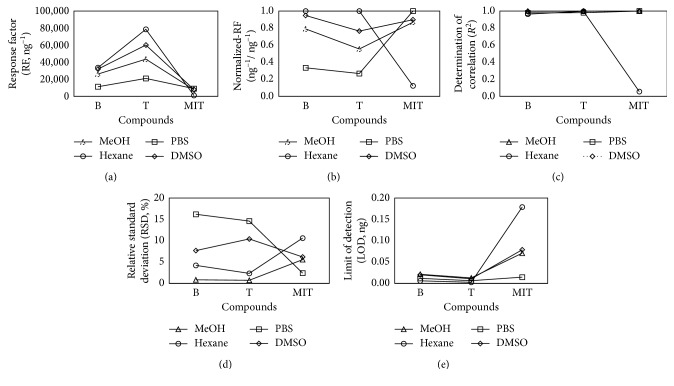
Plots of the calibration results from the three target compounds (B, T, and MIT) according to the four different solvents. (a) Response factor. (b) Normalized-RF. (c) Determination of correlation. (d) Relative standard deviation. (e) Limit of detection.

In order to conduct a toxicity testing, one needs to be able to obtain reliable quantitation data of the target toxic compounds. In this study, we assessed the effect of the solvent type on the quantitative results by analyzing three toxic compounds using four different solvents. Benzene, toluene, and MIT, which are well-known toxic compounds, were selected as target analytes. Liquid working standards of the target analytes were prepared using four different solvents (MeOH, hexane, PBS, and DMSO), which are commonly used for extraction and dilution of sample solutions. These working standards were analyzed using GC-MS, thereby providing the calibration results of the target compounds according to the solvent type. The solvent effect was then assessed by comparing these results (Figure 3). The RF values of nonpolar compounds (benzene and toluene) were the highest (33,674 ng^−1^ (benzene) and 78,604 ng^−1^ (toluene)) in nonpolar solvents such as hexane and the lowest (11,286 ng^−1^ (benzene) and 21,026 ng^−1^ (toluene)) in the polar solvent such as PBS. Unlike benzene and toluene, MIT had the highest RF value (9,067 ng^−1^) in a polar solvent (PBS), while it dropped dramatically to 1,117 ng^−1^ in a nonpolar solvent (hexane). Additionally, in MeOH, all target compounds showed fairly good reproducibilities with RSDs below 6% and linearity above 0.99. In contrast, hexane induced a low *R*^2^ value of MIT (0.0562), and PBS led to high RSD values of benzene and toluene (above 14%).

All in all, the results of this study confirmed that the quantitative results were affected by the solvent effect. Since quantitative results can differ depending on the solvent type, it is important to select the solvent by considering the physicochemical properties (i.e., polarity) of the target compounds. In addition, the use of different solvents in the quantitative analysis, such as in the extraction and dilution processes, could lead to difficulties in obtaining reliable quantitative data.

## Figures and Tables

**Table 1:** Basic information on target compounds and solvents.

Chemical group	Full name	Short name	Molecular formula	Molecular weight (g·mol^−1^)	Density (g·mL^−1^)	m/z^a^	CAS number	Chemical structure
Target compound	Benzene	—	C_6_H_6_	78.11	0.874	78	71-43-2	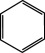
	Toluene	—	C_6_H_5_CH_3_	92.141	0.867	91	50643-04-4	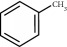
	Methylisothiazolinone	MIT	C_4_H_5_NOS	115.1	1.35	115	2682-20-4	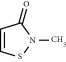

Solvent compound	Methanol	MeOH	CH_3_OH	32.04	0.792	31	67-56-1	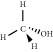
	Hexane	—	C_6_H_14_	86.18	0.6606	57	110-54-3	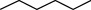
	Phosphate buffered saline	PBS	Cl_2_H_3_K_2_Na_3_O_8_P_2_	411.029	0.0648	NA	NA	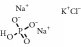
	Dimethyl sulfoxide	DMSO	C_2_H_6_OS	78.13	1.1004	45,63,78	67-68-5	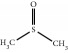

^a^Main spectra of the target compounds. NA, not available.

**Table 2:** Preparation of working standards (WSs) containing three target compounds (benzene, toluene, and MIT) based on four different solvents (MeOH, DMSO, hexane, and PBS).

*(a) Reagent grade chemical (RGC)*						
Compound name	Benzene	Toluene	MIT			
Concentration (%)	99.5	99.5	95			
Density (g·mL^−1^)	0.878	0.867	1.35			

*(b-1) The first primary standard (PS-1)*						
Compound	Benzene	Toluene	MeOH			
Volume (*μ*L)	20	20	1,960			
Dilution fraction	0.010	0.010				
Concentration (ng·*μ*L^−1^)	8,736	8,627				

*(b-2) The second primary standard (PS-2)*						
Compound	MIT					
Mass (mg)	180					
Volume (mL)	2.000					
Concentration (ng·*μ*L^−1^)	90,000					

*(c) The first working standard (WS)*						
Working standard	PS-1		PS-2	Solvent^a^		
Compound	Benzene	Toluene	MIT			
Volume (*μ*L)	100		100	1,800		
Dilution fraction	0.05		0.05			
Concentration (ng·*μ*L^−1^)	416	411	4,286			

*(d) The final working standard at 5 concentration levels*						
Order	Mixing volume (*μ*L)		Dilution fraction	Concentration (ng·*μ*L^−1^)		
	1st L-WS	Solvent		Benzene	Toluene	MIT
1	40	1,960	0.020	8.32	8.22	85.7
2	100	1,900	0.050	20.8	20.5	214
3	200	1,800	0.100	41.6	41.1	429
4	400	1,600	0.20	83.2	82.2	857
5	1,000	1,000	0.50	208	205	2,143

^a^Four solvents were used in this study: (1) MeOH, (2) DMSO, (3) hexane, and (4) PBS.

**Table 3:** Instrumental setup for the analysis of target compounds (B, T, and MIT).

*(a) Carrier gas settings*		
Injection temperature	250	°C
Injection mode	Split	
Carrier gas	Helium (>99.999%)	
Pressure	132.9	kPa
Column flow	2.41	mL·min^−1^ (constant flow)
Purge flow	3.0	mL·min^−1^
Split ratio	20	

*(b) Gas chromatography (model: GC-2010, Shimadzu, Japan)*		
Column	Rtx-5MS (Shimadzu, Japan) (length: 60 m, diameter: 0.25 nm, and film thickness: 0.25 *μ*m)	
Oven setting	40°C (4 min) ⟶ 145°C (15°C/min) ⟶ 285°C (70°C/min) (Total program time = 13 min)	

*(c) Mass spectrometry (model: GCMS-QP2010 ultra, Shimadzu, Japan)*		
Ionization mode	EI (70 eV)	
Ion source temperature	250	°C
Interface temperature	250	°C
TIC	30∼500	*m/z*
Scan speed	1000	

**Table 4:** Experimental results for three target compounds (benzene, toluene, and MIT) based on four different solvents (MeOH, hexane, PBS, and DMSO), including the response factor (RF, ng^−1^), normalized-RF (N-RF, ng^−1^/ng^−1^), determination of correlation (*R*^2^), relative standard deviation (RSD, %), and limit of detection (LOD, ng).

Solvent	Factors	Target compound		
		Benzene	Toluene	MIT
MeOH	RF (ng^−1^)	26,164	43,618	7,877
	N-RF^a^ (ng^−1^/ng^−1^)	0.79	0.55	0.87
	*R* ^2^	0.9978	0.9984	0.9994
	RSD (%)	0.83	0.72	5.56
	LOD (ng)	0.02	0.01	0.07

Hexane	RF (ng^−1^)	33,674	78,604	1,117
	N-RF (ng^−1^/ng^−1^)	1	1	0.12
	*R* ^2^	0.9630	0.9969	0.0562
	RSD (%)	4.22	2.35	10.6
	LOD (ng)	0.01	0.00	0.18

PBS	RF (ng^−1^)	11,286	21,026	9,067
	N-RF (ng^−1^/ng^−1^)	0.34	0.27	1
	*R* ^2^	0.9788	0.9801	0.9997
	RSD (%)	16.2	14.6	2.41
	LOD (ng)	0.01	0.01	0.01

DMSO	RF (ng^−1^)	31,932	60,147	8,148
	N-RF (ng^−1^/ng^−1^)	0.95	0.77	0.90
	*R* ^2^	0.9984	0.9995	0.9998
	RSD (%)	7.69	10.4	6.19
	LOD (ng)	0.02	0.01	0.08

^a^Normalized-RF (N-RF): RF value/maximum RF among four different solvents.

**Table 5:** List of the comparison of the solvent effects on chemical and biological analysis using the analytical instrument.

Order	Field of science	Target compound or material	Pretreatment or standard solvent	Sample solvent	Instrument or assay method^a^	Reference
1	Chemistry	13 aldehydes and 4 ketones	Water and acetonitrile	Water and methanol	HPLC-UV	Brandão et al. [23]
2		Formaldehyde in bovine milk	Ultrapure water	Acetonitrile	HPLC-UV	Rezende et al. [24]
3		Sodium ferrocyanide in 801 Salt	0.02 M NaOH	0.02 M NaOH	HPLC-UV	Lim et al. [25]

4	Biology	*Ginkgo biloba* L. (EGB)	Methanol	Isopropanol-ethanol-water (3 : 2 : 1)	HPLC-UV/DAD/MS	Yang et al. [26]
5		Vitamin C (ascorbic acid and dehydroascorbic acid)	10% meta-phosphoric acid	10% meta-phosphoric acid	HPLC or UPLC	Klimczak & Gliszczyńska-Świgło [27]
6		Sugars content in sunflower oil	0.005 N H_2_SO_4_	Ethanol and distilled water	HPLC	Baumler et al. [28]
7		37 raw vegetables	Acetone, methanol, ethanol, and distilled water	2,2-Diphenyl-1-picrylhydrazyl (DPPH) in ethanol	DPPH free radical scavenging assay	Sulaiman et al. [29]
				Distilled water	Total phenolic content	

^a^HPLC, high-performance liquid chromatography; UV, ultraviolet/visible; DVD, diode array detection; UPLC, ultraperformance liquid chromatography.

## Data Availability

The data used to support the findings of this study are included within the article.
